# Transmyometrial embryo transfer as a useful method to overcome difficult embryo transfers - a single-center retrospective study

**DOI:** 10.5935/1518-0557.20180029

**Published:** 2018

**Authors:** Janisse Ferreri dos Anjos, Emma Gabriela Portillo Osorio, Joana Peñarrubia, Ester Vidal, Francisco Fábregues Gasol

**Affiliations:** 1 Institut Clinic de Ginecologia, Obstetricia y Neonatología (ICGON). Hospital Clinic de Barcelona. Institut de Investigacions Biomédiques August Pi i Sunyer (IDIBAPS).

**Keywords:** Assisted reproductive technology (ART), cervical stenosis, cervical atresia, embryo transfer (ET), transmyometrial embryo transfer (TMET), pregnancy rate

## Abstract

**Objective:**

Pregnancy after an embryo transfer depends largely on embryo quality, endometrial receptivity, and the technique used in the embryo transfer. Embryo transfers have been reported as inevitably traumatic and difficult for 5-7% of patients in assisted reproduction treatment. In these cases, transmyometrial embryo transfer should be considered as a suitable method to overcome difficult embryo transfers. The aim of this study was to report our experience with this technique and analyze its causes, results and complications.

**Methods:**

Since 1993, 39 women (40 cycles of assisted reproductive technology treatment) were submitted to transmyometrial embryo transfers in our center. The procedures were carried out as described by the Towako group.

**Results:**

The enrolled female patients had a mean age of 34 years and a mean baseline FSH level of 6.89 IU/mL. The median number of retrieved oocytes was 7.50 and a mean of 2.63 embryos were transferred. Implantation rate was 9.5%. With respect to clinical results, pregnancy and miscarriage rates were 25% and 30%, respectively. Since there were two twin pregnancies, the live birth rate was 22.5% (9/40). No major complications were reported.

**Conclusion:**

Transmyometrial embryo transfer can and should be an option in cases of difficult/impossible transcervical embryo transfer.

## INTRODUCTION

The technique of embryo transfer is crucial and requires great attention and careful thought. Pregnancy after an embryo transfer (ET) depends on a number of factors, including embryo quality, endometrial receptivity, and embryo transfer technique itself.

Historically, embryo transfer methods have received little attention and minimal data has been published on the subject. The reason for this is their apparent simplicity. Nevertheless, difficult transfer procedures occur frequently and have been shown to decrease pregnancy rates significantly. It has been reported that embryo transfer is inevitably traumatic and difficult to 5-7% of patients in assisted reproduction treatment.(Tur-Kaspa *et al.*, 1998; Wood *et al*., 1985). Moreover, in about one percent of the cases the transcervical route may be nearly impossible to use, even by experienced practitioners, mainly due to anatomical and pathological cervical disorders such as congenital stenosis, atresia or previous trachelectomy (Healy *et al.*, 2015; Wood *et al.*, 1985).

Physicians facing such scenario might have one of the following options: (i) carry on and perform the various maneuvers available and thus experience a very difficult and traumatic transcervical embryo transfer (TCET); (ii) call off the fresh embryo transfer and perform a frozen embryo transfer at a later occasion after cervical dilatation (with or without hysteroscopy), hoping it will alleviate the difficulty; or (iii) attempt a transmyometrial embryo transfer (TMET) if the other options failed. Although applicable to only a few cases, the transmyometrial approach might be considered as a possibility for performing embryo transfers in cases where transcervical embryo transfer is very difficult to perform.

The aim of this study was to report our experience with this technique and analyze its causes, results and complications.

## MATERIALS AND METHODS

Since 1993, 39 women (40 cycles of assisted reproductive technology treatment) were offered transmyometrial embryo transfers in a tertiary ART clinic (FIV Clinic) in Barcelona, Spain. The standard clinical protocol in effect at the clinic contemplates TMET as the fifth alternative step for difficult/impossible transcervical embryo transfers.

TMET was performed mainly because transcervical embryo transfer (TCET) was an unviable option due to unmanageable cervical stenosis (37/39 patients) or other cervical anatomy abnormalities (2/39).

All patients but one received controlled ovarian stimulation with recombinant FSH injections (Gonal F Merck SL). In 35/39 women, a long agonist protocol was used to achieve ovarian stimulation. Antagonist protocol was prescribed to three patients. One case came from a natural cycle.

Oocyte maturation in the included patients was triggered by a single dose of recombinant human chorionic gonadotropin (r-HCG) (Ovitrelle, 250mcg, Merck SL), based on established estradiol levels and follicular diameters (leading follicle size of 18mm). Thirty-four to 36 hours later, transvaginal ultrasound-guided follicle aspiration (5MHz transvaginal probe, Aloka) was performed with the patients under anesthesia. Daily vaginal micronized progesterone (Utrogestan, SEID Lab) as luteal support was initiated for all patients from the day of oocyte retrieval.

With respect to embryo transfer, the same protocol has been followed in the clinic since 1993. Embryo transfer is usually performed transcervically with a flexible catheter (Flexible Cook Catheter / Flexible Wallace Catheter). Initially a soft embryo transfer catheter is used because there is good evidence (Grade A) indicating it improves IVF embryo transfer pregnancy rates (Practice Committee of the American Society for Reproductive Medicine, 2017). When ET is not possible, a consistent inner guide is introduced into the catheter to facilitate entrance into the external cervical os. When the cervix cannot be bypassed, a Pozzi tenaculum forceps is used to optimize cervix traction. If these three maneuvers fail, a fourth and last attempt to transfer embryos transcervically is performed through mechanical cervical dilatation. In this last step, complications such as uterine perforation or creation of false cervical passages must be considered. When these four attempts are unsuccessful, TMET is indicated.

When the patient is suspected for cervical distortion or stenosis, a mock ET at the time of oocyte retrieval is often performed. The method described by Coroleu *et al*. (2000) for ultrasound-guided transcervical intrauterine transfer has become the gold standard. Visualization has been shown to improve outcomes in transcervical ET. Ultrasound guidance allows accurate assessment of catheter position, thus helping physicians lead their way into the endometrial cavity. Cases of failed transcervical embryo transfer decreased significantly, along with the need for TMET.

**Figure 1:**
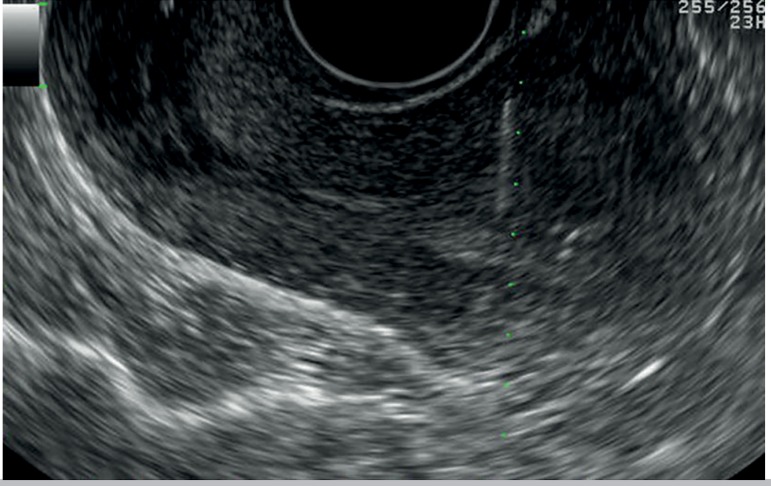
Transmyometrial ET procedure. The needle is inserted across the anterior myometrial wall aiming the endometrium stripe

**Figure 2:**
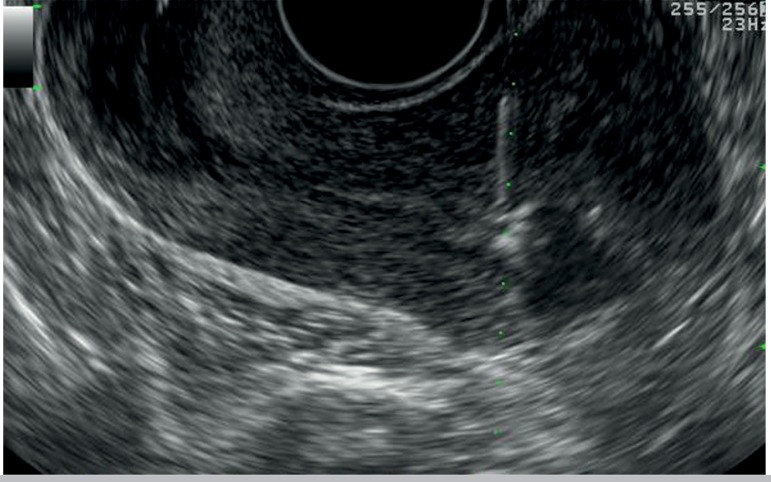
Transmyometrial ET procedure. Embryos suspended in culture medium are gently injected in the endometrial cavity approximately 1.5 cm from the fundus of the uterus. Embryo transfer is confirmed by a flow of echogenic fluid clearly seen inside the uterine cavity

In our center, transmyometrial embryo transfer is performed as described by Kato *et al*. (1993), with the Towako transfer set (Towako^®^ needle, Cook, USA) (Cook Medical, 2010). The procedure is performed with the patient sedated and with an empty bladder in dorsal lithotomy position. In the procedure, a transvaginal ultrasound probe with a Towako needle attached to the needle holder of the vaginal probe is used (Healy *et al*., 2015). Under direct visualization the uterus is scanned until the endometrial stripe is found; then the needle is inserted transmyometrially until it reaches the outer layer of the endometrium at the level of the uterine fundus (Kato *et al*., 1993) (Figure 1). An embryologist then loads the embryo suspended in culture medium with the inner catheter and inserted into the needle in a way that the catheter protrudes 1mm beyond the tip of the needle. The embryo is gently injected inside the endometrial cavity approximately 1.5cm from the fundus of the uterus; in cases of retroverted uterus, the needle may be inserted through the posterior fornix, posterior uterine wall or posterior endometrial border (Khairy *et al*., 2016; Akhtar *et al*., 2015). The embryo transfer is confirmed by a flow of echogenic fluid clearly seen inside the endometrial cavity (Figure 2). Finally, an embryologist checks whether the catheter still holds embryos before discarding it.

The patients rest for approximately two hours until they are completely recovered, and are then discharged to go home on the same day of the procedure.

## RESULTS

Baseline characteristics are described in Table 1. The enrolled female patients had a mean age of 34 years and a mean baseline FSH level of 6.89 IU/mL.

**Table 1:** Clinical characteristics and IVF outcomes for patients offered transmyometrial embryo transfer (TMET).

**Clinical Characteristics**		
Age (years)*	34	31.5-35
Baseline FSH (UI/L)	6.89	6.06-7.72
**Causes of infertility**		
Unexplained (n,%)	13	32.5
Male factor (n,%)	11	27.5
Endometriosis (n,%)	8	20.0
Tubal factor (n,%)	5	12.5
Donor sperm (n,%)	3	7.5
**Clinical results**		
Nº of oocytes retrieved†	7.5	5.25-11.75
Nº of embryos transferred*	2.63	2.42-2.83
Implantation rate (n,%)	10/105	9.5
Clinical pregnancy rate (n,%)	10/40	25
Miscarriage rate (n,%)	3/10	30
Live birth rate (n,%)	9/40	22.5

* Results are shown as mean and 95% CI.

† Results are shown as median and p25-p75 interquartile range.

Unexplained infertility (32.5%) was the leading cause of couple infertility, followed by male factor (27.5%), endometriosis (20%), tubal factor (12.5%), and donor sperm (7.5%).

With respect to ART outcomes, the median number of retrieved oocytes was 7.50, and a mean of 2.63 embryos were transferred.

Forty TMET procedures have been performed in our center since 1993, yielding an implantation rate of 9.5% (ten pregnancies from 105 transferred embryos). In terms of clinical outcomes, the pregnancy rate was 25% (10/40). Unfortunately, three pregnancies ended in miscarriage (30%). Since there were two twin pregnancies, the live birth rate was 22.5% (9/40).

TMET is a relatively quick and easy procedure to perform. Pain, bleeding, infection and injuries to adjacent organs are possible complications. No major complications were reported in our study. However, managing missed abortions may be somewhat complicated in these patients. Cervical dilatation and uterus aspiration might not be easily performed, and alternative ways to approach missed abortions must be pursued by clinicians.

During the first 10 years of the studied period, TMET accounted for approximately 7.8% (28 TMET) of all embryo transfers (n=3587) in our center. As described above, after the publication by Coroleu *et al*. (2000) ultrasound-guided transcervical embryo transfer became the gold standard, driving down the use of TMET to 1.2% (12/9534).

**Figure 3:**
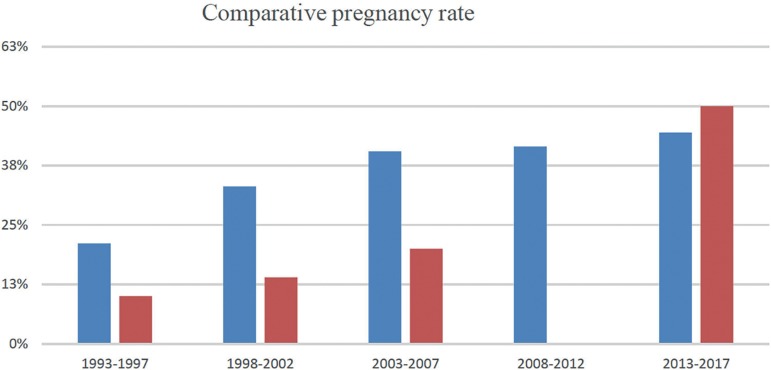
Comparative pregnancy rate between general Clinical Pregnancy Rate (blue columns) and Clinical Pregnancy Rate by TMET (red columns)

The analysis of clinical outcomes revealed an improvement in pregnancy rates by TMET throughout a 25-year observation period, despite the drop in the use of the procedure. An analysis of five-year periods (Figure 3) showed that the clinical pregnancy rate of TMET increased from 10% (1993-1997) to 14% (1998-2002), 20% (2008-2012), and 50% (2013-2017). This last clinical pregnancy rate (50%) was calculated from very few TMET procedures (n=4). No TMET procedure was performed from 2008 to 2012.

These findings were consistent with the progression of clinical pregnancy rates observed in our center during the studied period, as discussed below.

## DISCUSSION

Since the first pregnancy using IVF was achieved nearly 30 years ago, many features of the procedure have been significantly changed. In contrast, embryo transfer has remained relatively unaltered.

Today, one of the most challenging issues arising from ET involves the management of very difficult or impossible transcervical embryo transfer (TCET) procedures. Since there is no consensus over what constitutes a difficult ET, an accurate comparison of studies becomes even more troublesome (Phillips *et al.*, 2013; Akhtar *et al*., 2015). Nonetheless, it has been shown that the clinical pregnancy rate decreases progressively as additional maneuvers are performed during ET (Kava-Braverman *et al*., 2017). An alternative technique for embryo transfer that bypasses the cervical canal is the Towako method, otherwise known as TMET. This is potentially a good option for patients with severe cervical stenosis or history of difficult embryo transfers.

Transmyometrial embryo transfers have been reported in 15 studies: 11 case reports, 3 case series, and one randomized clinical trial (Khairy *et al*., 2016; Huberlant *et al*., 2014; Muñoz *et al*., 2014; Sullivan-Pyke *et al*., 2014; Lin *et al.*, 2010; Jamal *et al*., 2009; Ohl *et al*., 2009; Xu *et al*., 2009; Lai *et al*., 2001; Anttila *et al*., 1999; Lesny *et al*., 1999; Asaad & Carver-Ward, 1997; Groutz *et al*., 1997; Sharif *et al*., 1996; Kato *et al*., 1993). The results reported in these studies are controversial because the inclusion criteria are heterogeneous. Even though TMET was performed in cases of difficult conventional ET, it is hard to accurately describe the degree of difficulty encountered in the procedure. This might explain the discordant clinical pregnancy rates published in these studies.

As mentioned above, several aspects of a difficult TCET may reduce the clinical pregnancy rate, including endometrial injury or the induction of uterine contractions. Physicians have the option to carry on with the TCET procedure progressively with additional maneuvers, knowing that the pregnancy rate might decrease with each maneuver, or patients may be offered to proceed with embryo freezing (or re-freezing) and then have an optimized TCET using the cervical approach or with anesthesia before reattempting the TCET. Further studies are required to compare those approaches.

In our study the clinical pregnancy rate was lower (25%) when compared to the 32% reported by Khairy *et al*. (2016) and the 36.5% by Kato *et al*. (1993). However, we must point out that the poorer outcomes seen in our series might be explained by the systematic use of additional maneuvers before TMET (Pasqualini & Quintans, 2001; Ghazzawi *et al.,* 1999). One study reported that TMET led to increased junctional zone contractions, which is believed to decrease the chance of implantation. However, the same study showed that very difficult embryo transfers also triggered zone contractions with similar frequency and amplitude (Biervliet *et al*., 2002).

The use of ultrasound guidance in embryo transfers started in 2001, leading to a decrease in the number of TMET procedures. Nearly two thirds (67.5%) of our cases were performed prior to the use of ultrasound guidance during the embryo transfer. The strengths of this study lie in the number of cases included - all of which from the same center - and the homogeneous inclusion criteria adopted, according to which all TMET procedures were performed after the same sequence of maneuvers. On the other hand, a weakness is the retrospective nature of our cases, many of which are not very recent.

Although suitable for a few cases, TMET might be considered as a viable option in cases where TCET is difficult to perform. TMET is not a novel option, but it should be thought of as a useful approach to help women with troublesome conventional embryo transfers. Even though improvements in catheters and embryo transfer conditions with the use of hysteroscopy have relegated TMET to a lesser position, the procedure might still be useful in certain cases.

## CONCLUSION

This retrospective single-center study summarized our 25-year experience with TMET. Our results reflect the cases of a group of patients with great difficulty in conventional ET, in which the same maneuvers were performed before proceeding to TMET. The results published herein might have been better if TMET had been performed earlier to minimize the traumatic effect of other maneuvers.
